# Supplementary material for: The Shortest Duration Constrained Hidden Markov Model: Data denoise and forecast optimization on the country-product matrix for the Fitness-Complexity Algorithm
Source: PLoS One. 2021 Jul 26;16(7):e0253845. doi: 10.1371/journal.pone.0253845 (PMC8312928; doi:10.1371/journal.pone.0253845)
Supplement: S1 Appendix — (DOCX) [file pone.0253845.s002.docx]

## Appendix

**Appendix A: Time-Divided Robustness Test**

This section presents the method of time-divided regression on the model of Test 4: Fitness-GDP Panel Regression Test. We divide the period into 1995-2007 and 2008-2017 and perform regression analysis in these two periods respectively (Appendix A1-4) .

Tables A1 and A2 show the test results of SDC-HMM noise reduction from 1995-2007 and 2008-2017 respectively. Tables A3 and A4 show the test results of HMM noise reduction from 1995-2007 and 2008-2017 respectively. Considering the significance level of core variables, the results show that original model results are robust. Taking the 2008 financial crisis as the separation point, the time-divided regression can not only test the robustness of the model, but also test how the correlation between economic fitness to GDP growth changed before and after the financial crisis.

Table A1 Regression results of mixed regression model and fixed effect model of economic fitness for GDP prediction after SDC-HMM noise reduction from 1995-2007.

| log** | Model 1 | Model 2 | Model 3 | Model 4 | Model 5 | Model 6 |
| --- | --- | --- | --- | --- | --- | --- |
| lnFitness | -.0178  (0.974) | 1.0653***  (0.002) | .4831*  (0.097) | .8979**  (0.029) | .4684  (0.290) | .2072  (0.623) |
| lnPop |  | -.5934***  (0.000) | -2.9163***  (0.000) |  | 2.6920**  (0.040) | -6.4904*  (0.056) |
| lnLF |  |  | 2.2210***  (0.000) |  |  | 6.4496***  (0.005) |
| Cons | 7.6093***  (0.000) | 18.052***  (0.000) | 22.416***  (0.000) | 6.6887***  (0.000) | -45.202*  (0.073) | 13.3943  (0.661) |
| $R^{2}$ | -0.0172 | 0.6393 | 0.7704 | 0.0851 | 0.1555 | 0.2777 |
| obs | 60 | 60 | 60 | 60 | 60 | 60 |
| F-statistics | 0.00 | 53.28 | 67.00 | 5.02 | 4.88 | 6.66 |
| P-statistics | 0.9736 | 0.0000 | 0.0000 | 0.0292 | 0.0114 | 0.0007 |
| F-test | - | - | - | 66.23 | 16.29 | 8.19 |
| Prob>F | - | - | - | 0.0000 | 0.0000 | 0.0000 |

Notes: * p < 0.1, ** p < 0.05, *** p < 0.01; $R^{2}$ is adjusted for Models 1 to 3, and their significance is tested with an F-statistic; $R^{2}$ is the overall value (top line) and the within value (bottom line) for Models 4 to 6, and their significance is tested with the Wald Chi statistic.

| log** | Model 1 | Model 2 | Model 3 | Model 4 | Model 5 | Model 6 |
| --- | --- | --- | --- | --- | --- | --- |
| lnFitness | .0922  (0.784) | 1.0328***  (0.001) | .2483*  (0.076) | .8979**  (0.029) | .4684  (0.290) | .8609***  (0.005) |
| lnPop |  | -.4872***  (0.000) | -3.6981***  (0.000) |  | 2.6920**  (0.040) | 6.7326***  (0.009) |
| lnLF |  |  | 3.2265***  (0.000) |  |  | -4.7861**  (0.030) |
| Cons | 8.5983***  (0.000) | 17.079***  (0.000) | 20.148***  (0.000) | 6.6887***  (0.000) | -45.202*  (0.073) | -33.997  (0.104) |
| $R^{2}$ | 0.0018 | 0.4613 | 0.8995 | 0.0851 | 0.1555 | 0.3848 |
| obs | 45 | 45 | 45 | 45 | 45 | 45 |
| F-statistics | 0.08 | 19.84 | 132.31 | 5.02 | 4.88 | 7.72 |
| P-statistics | 0.7842 | 0.0000 | 0.0000 | 0.0292 | 0.0114 | 0.0004 |
| F-test | - | - | - | 66.23 | 16.29 | 11.98 |
| Prob>F | - | - | - | 0.0000 | 0.0000 | 0.0000 |

Table A2 Regression results of mixed regression model and fixed effect model of economic fitness for GDP prediction after SDC-HMM noise reduction from 2008-2017.

Notes: * p < 0.1, ** p < 0.05, *** p < 0.01; $R^{2}$ is adjusted for Models 1 to 3, and their significance is tested with an F-statistic; $R^{2}$ is the overall value (top line) and the within value (bottom line) for Models 4 to 6, and their significance is tested with the Wald Chi statistic.

Table A3 Regression results of mixed regression model and fixed effect model of economic fitness for GDP prediction after HMM noise reduction from 1995-2007.

| ln** | Model 1 | Model 2 | Model 3 | Model 4 | Model 5 | Model 6 |
| --- | --- | --- | --- | --- | --- | --- |
| lnFitness | -.1936  (0.721) | 1.0719***  (0.003) | .4709  (0.116) | .7746*  (0.058) | .3294  (0.451) | .0764  (0.854) |
| lnPop |  | -.6015***  (0.000) | -2.9301***  (0.000) |  | 2.8855**  (0.028) | -6.5256*  (0.055) |
| lnLF |  |  | 2.2315***  (0.000) |  |  | 6.5943***  (0.004) |
| Cons | 7.7892***  (0.000) | 18.186***  (0.000) | 22.495***  (0.000) | 6.7997***  (0.000) | -48.829*  (0.053) | 11.5137  (0.706) |
| $R^{2}$ | -0.0150 | 0.6370 | 0.7693 | 0.0648 | 0.1466 | 0.2747 |
| obs | 60 | 60 | 60 | 60 | 60 | 60 |
| F-statistics | 0.13 | 52.76 | 66.58 | 3.74 | 4.55 | 6.57 |
| P-statistics | 0.7214 | 0.0000 | 0.0000 | 0.0584 | 0.0150 | 0.0008 |
| F-test | - | - | - | 64.32 | 16.17 | 8.12 |
| Prob>F | - | - | - | 0.0000 | 0.0000 | 0.0000 |

Notes: * p < 0.1, ** p < 0.05, *** p < 0.01; $R^{2}$ is adjusted for Models 1 to 3, and their significance is tested with an F-statistic; $R^{2}$ is the overall value (top line) and the within value (bottom line) for Models 4 to 6, and their significance is tested with the Wald Chi statistic.

Table A4 Regression results of mixed regression model and fixed effect model of economic fitness for GDP prediction after HMM noise reduction from 2008-2017.

| ln** | Model 1 | Model 2 | Model 3 | Model 4 | Model 5 | Model 6 |
| --- | --- | --- | --- | --- | --- | --- |
| lnFitness | .0349  (0.925) | 1.0769***  (0.002) | .2494  (0.104) | 1.0696***  (0.009) | 1.1492***  (0.005) | .8538**  (0.031) |
| lnPop |  | -.4819***  (0.000) | -3.7229***  (0.000) |  | 1.9948*  (0.098) | 7.5001***  (0.005) |
| lnLF |  |  | 3.2530***  (0.000) |  |  | -5.3581**  (0.020) |
| Cons | 8.6629***  (0.000) | 16.970***  (0.000) | 20.143***  (0.000) | 7.5638***  (0.000) | -30.516  (0.182) | -38.407*  (0.080) |
| $R^{2}$ | -0.0230 | 0.4420 | 0.8982 | 0.1623 | 0.2213 | 0.3290 |
| obs | 45 | 45 | 45 | 45 | 45 | 45 |
| F-statistics | 0.01 | 18.43 | 130.46 | 7.56 | 5.40 | 6.05 |
| P-statistics | 0.9250 | 0.0000 | 0.0000 | 0.0090 | 0.0086 | 0.0019 |
| F-test | - | - | - | 165.75 | 88.49 | 10.47 |
| Prob>F | - | - | - | 0.0000 | 0.0000 | 0.0000 |

Notes: * p < 0.1, ** p < 0.05, *** p < 0.01; $R^{2}$ is adjusted for Models 1 to 3, and their significance is tested with an F-statistic; $R^{2}$ is the overall value (top line) and the within value (bottom line) for Models 4 to 6, and their significance is tested with the Wald Chi statistic.
