# Supplementary material for: The Shortest Duration Constrained Hidden Markov Model: Data denoise and forecast optimization on the country-product matrix for the Fitness-Complexity Algorithm
Source: PLoS One. 2021 Jul 26;16(7):e0253845. doi: 10.1371/journal.pone.0253845 (PMC8312928; doi:10.1371/journal.pone.0253845)
Supplement: Attachment — Submitted filename: Report_PONE_D_20_26064R1.pdf [file pone.0253845.s004.pdf]

Report on the paper
"*The Shortest Duration Constrained Hidden Markov Model: Data Denoise and Forecast Optimization on the Country-Product Matrix for the Fitness-Complexity Algorithm*"

by Pengcheng Song, Xiangyu Zong, Ximing Chen and Qin Zhao.

The article applies Shortest Duration Constrained Hidden Markov Model (SDC-HMM) on data denoising for an economic complexity index. Based on export data during 1995-2017, the authors use SDC-HMM to optimize the forecasting of GDP trend.

I find that the techniques and results are interesting. I wish that some of the details are further clarified and improved.

Comments

1. The keywords should reflect the main research areas. The two main effects of the SDC-HMM both should be involved. Please specify them clearly.

2. The introduction part is organized with order, but in order to give readers a better picture I suggest introducing more details of your approach i.e. SDC-HMM, and stressing the importance of it.

3. Page 13, the authors claim that "It takes about 10-20 minutes to perform the SDC-HMM algorithm of the noise reduction task in this paper by using a PC." The computational complexity should be further discussed, for example, how is it compared with previous literature?

4. Figures need brief notes and so for Tables in order to indicate the readers enough information. Please add essential notes for each table.

5. The discussion part should be further improved. The key findings, importance and limitations should be further discussed.

6. Please add a full stop or comma (if the sentence is not finished) symbol behind each equation.

Misprints

1. Page 13, Footnote 1, "mmodity" should be "Commodity".

2. Page 13, Footnote 2, "Band" should be "Bank".
