# Supplementary material for: The Shortest Duration Constrained Hidden Markov Model: Data denoise and forecast optimization on the country-product matrix for the Fitness-Complexity Algorithm
Source: PLoS One. 2021 Jul 26;16(7):e0253845. doi: 10.1371/journal.pone.0253845 (PMC8312928; doi:10.1371/journal.pone.0253845)
Supplement: Attachment — Submitted filename: response to reviewers.docx [file pone.0253845.s003.docx]

Thanks to your careful and valuable comments. We will explain your concerns point by point.

Reviewer #1:

Q1: What is the contribution of this paper? The innovation points of the research are not clear.\\

A1: The possible contributions of this paper are added in page 3, and we divide them into four different aspects. The main innovations of this paper are also added in page 3, and we divide them into two different aspects.

Q2: Why $X_2$ in the Hidden Markov Model is different from the other parameters?\\

A2: The $X_2$ is incorrectly capitalized, and we have corrected it in page 3 line 72.

Q3: How to choose the value of $d$? Does it have specific meaning in the economic field?\\

A3: The choice of this value of $d$ aims to improve the economic forecasting effect through two aspects, they are introduced in the Results. The value of $d$ has specific meaning in the economic field, the economic characteristics and implications are introduce in the Results.

Q4: Why can $d$ be set to improve the effect?\\

A4: The reason lies in the strong economic characteristics and implications of $d$ in economic time series data, please see in Results too.

Q5: The symbols do not change before and after standardization in page 5 line 157.\\

A5: The symbols after standardization are misspelled, and we have corrected them in page 5 line 157.

Q6: Most of the pictures are not sharp enough.\\

A6: Some pictures are modified to make them more sharp.

Q7: With the proposed method in this paper, the computational complexity of the algorithm needs to be analyzed.

A7: We briefly introduce the computational complexity of the algorithm in the Methods
