# Supplementary material for: The Shortest Duration Constrained Hidden Markov Model: Data denoise and forecast optimization on the country-product matrix for the Fitness-Complexity Algorithm
Source: PLoS One. 2021 Jul 26;16(7):e0253845. doi: 10.1371/journal.pone.0253845 (PMC8312928; doi:10.1371/journal.pone.0253845)
Supplement: Attachment — Submitted filename: Response to Reviewers.docx [file pone.0253845.s005.docx]

Thanks to your careful and valuable comments. We will explain your concerns point by point.

Q1: The keywords should reflect the main research areas. The two main effects of the SDC-HMM both should be involved. Please specify them clearly.

A1: The two main effects of the SDC-HMM both have been involved. The new keywords: country-product matrices; constrained Hidden Markov Model; data denoise; forecast optimization.

Q2: The introduction part is organized with order, but in order to give readers a better picture I suggest introducing more details of your approach i.e. SDC-HMM, and stressing the importance of it.

A2: We have added a detailed description of the SDC-HMM method and explained the importance of the SDC-HMM method in two aspects. First, setting a minimum duration greater than one period is conducive to making full use of data time dimension information and effectively removing abnormal data value noise. Second, setting the value of d to be greater than or equal to 2 is to fully retain the information of data time dimension and identify the change of data trend in time.

Q3: Page 13, the authors claim that “It takes about 10-20 minutes to perform the SDC-HMM algorithm of the noise reduction task in this paper by using a PC.” The computational complexity should be further discussed, for example, how is it compared with previous literature?

A3: We have further discussed the computational complexity. As most of the computation is applied in the parameters of HMM, the shortest duration constraint (SDC) only consumes a small amount of calculation. Compared with the calculation method of Tacchella et al. (2018), we provide a better noise reduction method without increasing the amount of calculation.

Q4: Figures need brief notes and so for Tables in order to indicate the readers enough information. Please add essential notes for each table.

A4: We have added brief but essential notes to all the figures and tables.

Q5: The discussion part should be further improved. The key findings, importance and limitations should be further discussed.

A5: The key findings, importance and limitations have been further discussed in the discussion part. we find that the SDC-HMM excels at removing abnormal high value (low value) noise in the stationary period, abnormal low value (high value) noise in the rising period and abnormal high value (low value) noise in the descending period, and in identifying the long-term change point of the data in low-to-high and high-to-low long-term trends, achieving obvious noise reduction. The limitation is for the shortest duration constraint is defined as a constant 2, which could be different in other economic fields.

Q6: Please add a full stop or comma (if the sentence is not finished) symbol behind each equation.

A6: A full stop or comma symbol has been added behind each equation, in which the sentence is not finished.

Misprints

Q1: Page 13, Footnote 1, “mmodity” should be “Commodity”.

A1: The misprint has been corrected.

Q2: Page 13, Footnote 2, “Band” should be “Bank”.

A2: The misprint has been corrected.
